# Crystal structures and Hirshfeld surfaces of differently substituted (*E*)-*N*′-benzyl­idene-*N*-methyl-2-(thio­phen-2-yl)acetohydrazides

**DOI:** 10.1107/S2056989017014384

**Published:** 2017-10-13

**Authors:** Laura N. F. Cardoso, Thais C. M. Noguiera, Carlos R. Kaiser, James L. Wardell, Marcus V. N. de Souza, William T. A. Harrison

**Affiliations:** a Fundação Oswaldo Cruz, Instituto de Tecnologia em Fármacos–FarManguinhos, Rua Sizenando Nabuco, 100, Manguinhos, 21041-250 Rio de Janeiro, Brazil; b Instituto de Química, Universidade Federal do Rio de Janeiro, Cidade Universitária, Rio de Janeiro, Brazil; c Department of Chemistry, University of Aberdeen, Meston Walk, Aberdeen AB24 3UE, Scotland

**Keywords:** crystal structure, carbohydrazide, methyl­ation, weak hydrogen bonds

## Abstract

The syntheses and crystal structures of (*E*)-*N*′-(3-cyano­benzyl­idene)-*N*-methyl-2-(thio­phen-2-yl)acetohydrazide, C_15_H_13_N_3_OS, (I), and (*E*)-*N*′-(4-meth­oxy­benzyl­idene)-*N*-methyl-2-(thio­phen-2-yl)acetohydrazide, C_15_H_16_N_2_O_2_S, (II), with different substituents in the *meta* and *para* position of the benzene ring are described. Compounds (I) and (II) both crystallize with two mol­ecules in the asymmetric unit, with generally similar conformations [r.m.s. overlay fits for (I) and (II) of 0.334 and 0.280 Å, respectively] that approximate to L-shapes. The thio­phene rings in (I) are well ordered, whereas those in (II) exhibit ‘flip’ rotational disorder [occupancies 0.662 (2) and 0.338 (2) for mol­ecule 1, and 0.549 (3) and 0.451 (3) for mol­ecule 2]. The packing for (I) features short C—H⋯O inter­actions arising from the C—H grouping adjacent to the cyanide group and C—H⋯N_c_ (c = cyanide) links arising from the methine groups to generate [110] double chains. Weak C—H⋯π inter­actions inter­link the chains into a three-dimensional network. The packing for (II) features numerous C—H⋯O and C—H⋯π inter­actions arising from different donor groups to generate a three-dimensional network. Hirshfeld fingerprint plots indicate significant differences in the percentage contact surfaces for (I) and (II).

## Abstract 2

The title compounds feature various types of weak inter­molecular inter­actions but their decomposed Hirshfeld fingerprint plots show significant differences.

## Chemical context   

Thio­phene derivatives are important heterocyclic compounds widely used as building blocks in many agrochemicals and pharmaceuticals (Swanston, 2006 ▸). A valuable group of thio­phenyl derivatives are the series of acyl­hydrazine derivatives, 2-[ArCH=N—N*R*CO(CH_2_)_*n*_-thio­phene, where *R* = Me or H, and *n* = 0 or 1. Recent studies have investigated their anti-TB activities (Cardoso *et al.*, 2014 ▸) and anti-cancer activities (Cardoso *et al.*, 2017 ▸). We now report the crystal structures of two derivatives of the 2-[ArCH=N—NMeCOCH_2_-thio­phene series, bearing different substituents at the *meta* and *para* positions of the benzene ring, *viz*. (*E*)-*N*′-(3-cyano­benzyl­idene)-*N*-methyl-2-(thio­phen-2-yl)acetohydrazide, (I), and (*E*)-*N*′-(4-meth­oxy­benzyl­idene)-*N*-methyl-2-(thio­phen-2-yl)acetohydrazide, (II). These complement our recent structural study (Cardoso *et al.*, 2016*a* ▸) of isomeric *ortho*-, *meta*- and *para*-nitro derivatives in the same family.

## Structural commentary   

**Figure 1:**
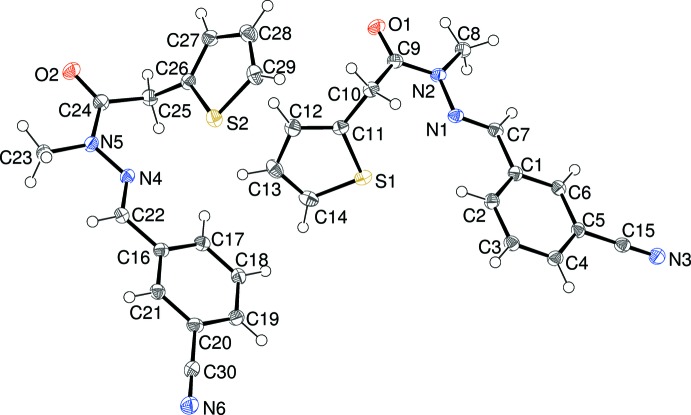
The mol­ecular structure of (I) showing 50% displacement ellipsoids.

**Figure 2:**
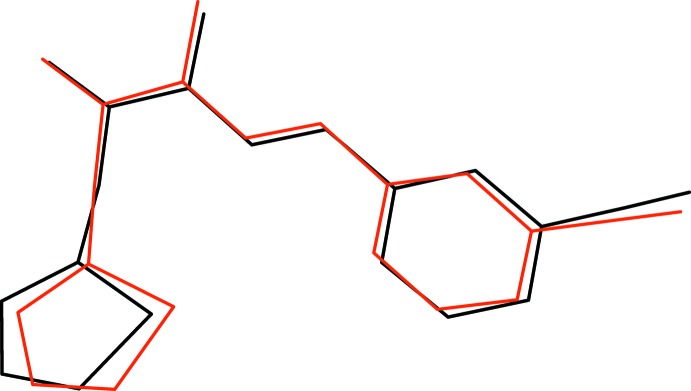
Overlay plot of mol­ecules *A* (red) and *B* (black) for (I).

The mol­ecular structure of (I) is shown in Fig. 1 ▸, which indicates the presence of two mol­ecules, *A* (containing S1) and *B* (containing S2), in the asymmetric unit of the triclinic unit cell. The thio­phene rings are well ordered [C11—S1—C14 = 92.14 (8); C26—S2—C29 = 92.39 (8)°]. For mol­ecule *A*, the dihedral angle between the thio­phene and benzene rings is 64.44 (5)°. The central CH=N—N(CH_3_)—C(=O) fragment (C7/C8/C9/N1/N2/O1) in (I) is almost planar (r.m.s. deviation = 0.022 Å) and subtends dihedral angles of 2.28 (9) and 66.47 (5)° with the benzene and thio­phene rings, respectively. The major twist in the mol­ecule occurs about the C9—C10 bond [N2—C9—C10—C11 = −91.98 (16)°], giving the mol­ecule an approximate overall L-shape. As seen for related compounds (Cardoso *et al.*, 2016*a* ▸), the N1—N2 bond length of 1.3797 (17) Å is significantly shortened compared to the reference value of ∼1.41 Å for an isolated N—N single bond and the C9—N2 amide bond of 1.3702 (19) Å is lengthened: these distance data can be inter­preted in terms of significant delocalization of electrons over the methyl­idene–acetohydrazide fragment of the mol­ecule. For mol­ecule *B*, comparable geometrical data are as follows: C16–C21 benzene ring = *A*, C26–C29/S2 thio­phene ring = *B*, C22/N4/N5/C23/C4/O2 linking chain (r.m.s. deviation = 0.033 Å) = *C*; dihedral angles *A*/*B*, *A*/*C* and *B*/*C* = 66.40 (8), 10.85 (9) and 58.33 (5)°, respectively; N5—C24—C25—C26 = −82.29 (18)°, N4—N5 = 1.3651 (17), C24—N5 = 1.3766 (19) Å. These data are generally similar to the corresponding values for mol­ecule *A* and the r.m.s. overlay fit of mol­ecules *A* and *B* of 0.334 Å and visual inspection (Fig. 2 ▸) confirms this.
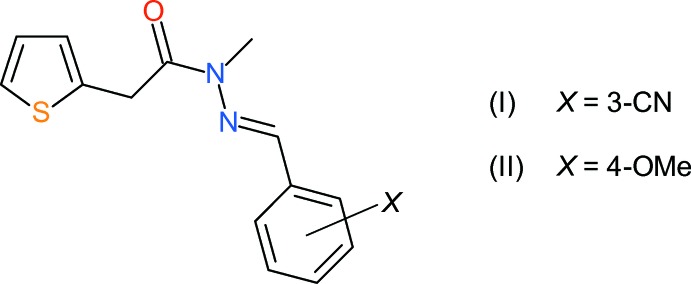


**Figure 3:**
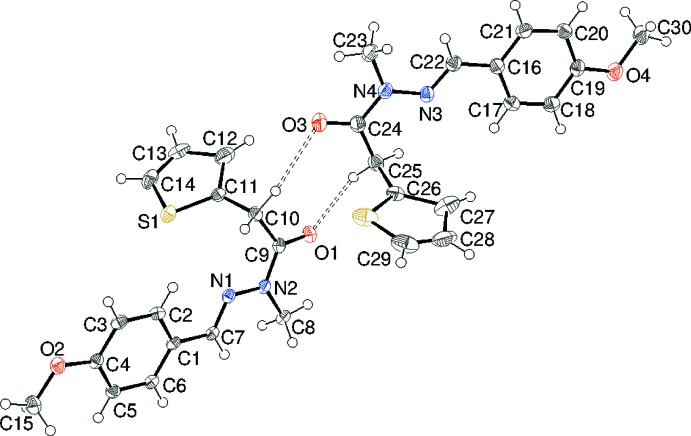
The mol­ecular structure of (II) showing 50% displacement ellipsoids. Only the major orientation of the thio­phene ring is shown.

**Figure 4:**
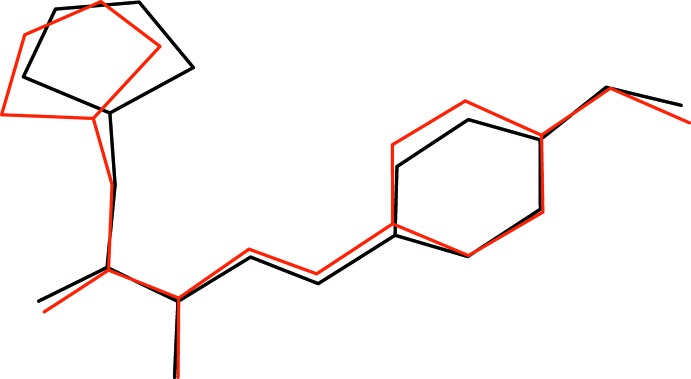
Overlay plot of mol­ecules *A* (red) and *B* (black) for (II). Only the major orientation of the thio­phene ring is shown.

Compound (II) (Fig. 3 ▸) also crystallizes in space group *P* 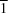 with *Z*′ = 2 (mol­ecule *A* containing S1 and mol­ecule *B* containing S2). In this case, both thio­phene ring show ‘flip’ disorder over two conformations rotated by ∼180° in a 0.662 (2):0.338 (2) ratio about the C10—C11 bond for *A* and a 0.549 (3):0.451 (3) ratio about the C25—C26 bond for *B*. The major orientation for *A* has the S atom pointing towards the benzene ring. For *B*, the disorder is close to statistical, but there is a slight preference for the S atom to point away from the benzene ring. For mol­ecule *A*, the dihedral angle between the thio­phene and benzene rings is 79.38 (7)°. The central CH=N—N(CH_3_)—C(=O) fragment (C7/C8/C9/N1/N2/O1) is almost planar (r.m.s. deviation = 0.013 Å) and the benzene and thio­phene rings are twisted from it by 0.89 (12) and 78.80 (9)°, respectively. Thus, as for (I), the major twist in the mol­ecule occurs about C9—C10 [N2—C9—C10—C11 = −86.1 (3)°], and an approximate overall L-shape results. Atom C15 of the meth­oxy group deviates slightly, by 0.068 (2) Å, from the plane of its attached ring. The N1—N2 [1.3780 (16) Å] and C9—N2 [1.3690 (18) Å] bond lengths show the same pattern as for (I), again indicating delocalization of electrons over the central grouping. Corresponding data for mol­ecule *B* in (II) are as follows: C16–C21 benzene ring = *A*, C26–C29/S2 thio­phene ring = *B*, C22/N3/N4/C23/C4/O3 linking chain (r.m.s. deviation = 0.021 Å) = *C*; dihedral angles *A*/*B*, *A*/*C* and *B*/*C* = 70.61 (8), 9.73 (17) and 77.66 (6)°, respectively; N4—C24—C25—C26 = 84.33 (17)°, N3—N4 = 1.3768 (17), C24—N4 = 1.375 (2) Å, displacement of C30 from the *A* ring = 0.155 (3) Å. Again, the two mol­ecules have broadly similar conformations (Fig. 4 ▸) and the r.m.s. overlay fit is 0.280 Å.

## Supra­molecular features   

Given that there are no classical donor groups, the packing motifs for (I) and (II) are dominated by a variety of non-classical C—H⋯O, C—H⋯N and C—H⋯S, C—H⋯π and π-π- inter­actions (Tables 1 ▸ and 2 ▸).

**Figure 5:**
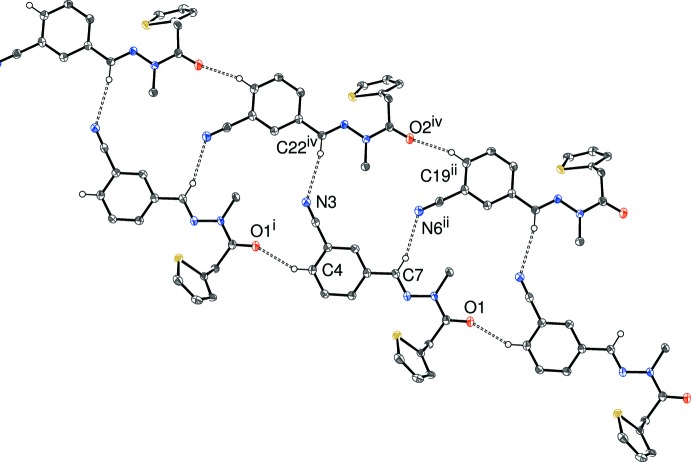
Fragment of a [110] hydrogen-bonded chain in the crystal of (I). Symmetry codes as in Table 1 ▸; additionally (iv) *x* + 1, *y*, *z* − 1. All hydrogen atoms not involved in hydrogen bonds omitted for clarity.

In (I), it is notable that both C—H groupings adjacent to the cyanide groups [i.e.:C4 (mol­ecule *A*) and C19 (mol­ecule *B*) in the 4-positions of the benzene rings] participate in short C—H⋯O inter­actions to generate separate [110] chains of *A* and *B* mol­ecules, both of which feature *C*(10) chain motifs, with adjacent mol­ecules in the chain related by translation symmetry. We may speculate that these C—H groupings have been ‘activated’ (made more acidic) by being adjacent to the electron-withdrawing cyanide group (Pedireddi & Desiraju, 1992 ▸). The chains are cross-linked by C—H⋯N hydrogen bonds: in each case the donor is the methine group [*i.e*. C7 (mol­ecule *A*) and C22 (mol­ecule *B*)] and the acceptor is the cyanide-N atom of the other asymmetric mol­ecule, *i.e. A* → *B* and *B* → *A*. This results in double chains (Fig. 5 ▸) propagating in [110] in which 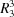
(18) loops are apparent. The chains are cross-linked by C—H⋯π inter­actions, with all the rings (*i.e*. both thio­phene and both benzene rings) acting as acceptors. The shortest centroid–centroid separation between aromatic rings is 3.9895 (10) Å, indicating that any π–π stacking effects in (I) are very weak at best.

The packing for (II) is less ‘tidy’ in the sense that C—H entities belonging to several different groups (benzene ring, methyl­ene group adjacent to the thio­phene ring, thio­phene ring, meth­oxy group) act as donors and none of the C—H⋯O links are particularly short. There are mol­ecule *A* → mol­ecule *A*, *A* → *B*, *B* → *A* and *B* → *B* links. Perhaps the most notable are a pair of bonds arising from the methyl­ene groups that generate *A* + *B* dimers incorporating 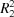
(8) loops, as shown in Fig. 3 ▸ above. A number of C—H⋯π inter­actions are observed, with all the rings acting as acceptors, but there are no aromatic π–π stacking inter­actions in (II) (shortest centroid–centroid separation > 4.9Å). When the different inter­molecular inter­actions are taken together, a three-dimensional network arises in the crystal of (II).

## Hirshfeld analysis   

**Figure 6:**
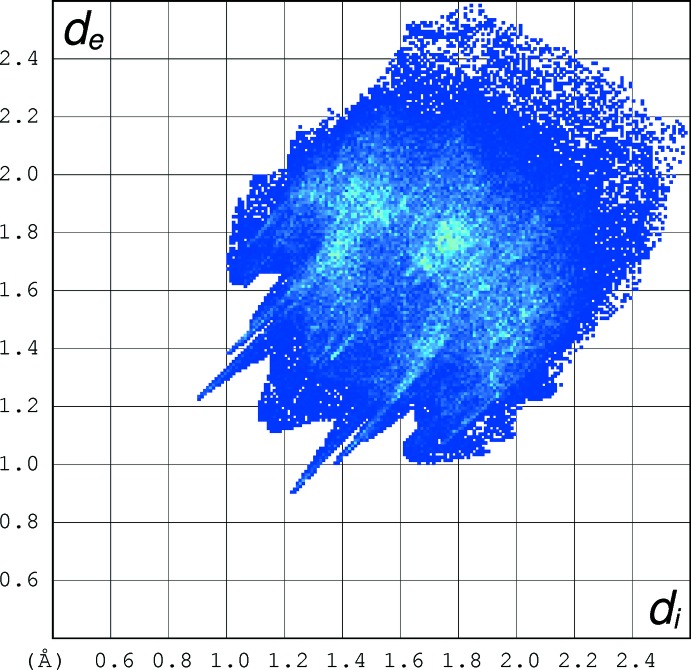
Hirshfeld fingerprint plot for (I)

**Figure 7:**
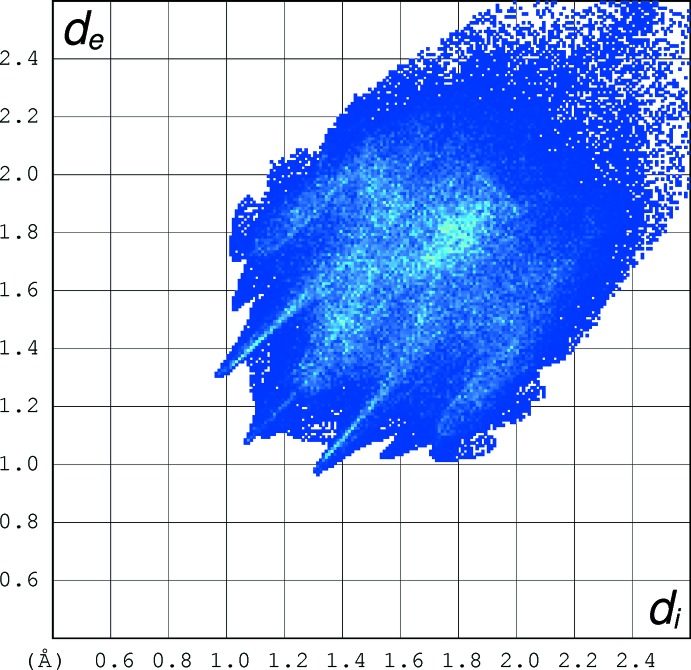
Hirshfeld fingerprint plot for (II)

Hirshfeld surface fingerprint plots for (I) (Fig. 6 ▸) and (II) (Fig. 7 ▸) were calculated with *CrystalExplorer17* (Turner *et al.*, 2017 ▸). The plot for (I) has ‘wingtip’ features that correspond to the short C—H⋯O hydrogen bonds described above, although the wingtips are not as pronounced as those seen for classical hydrogen bonds (compare: McKinnon *et al.*, 2007 ▸). In (II), the wingtips are less apparent, presumably reflecting the longer (and weaker) C—H⋯O inter­actions in this structure, even though there are more of them in (II) than in (I).

When the fingerprint plots for (I) and (II) are decomposed into the separate types of contacts (McKinnon *et al.*, 2007 ▸), some inter­esting differences arise (Table 3 ▸): H⋯H contacts represent the highest percentage in both structures, but they are far more significant in (II), representing over half the contact area, some 20% more than in (I). This deficit is largely made up by N⋯H/H⋯N contacts (*i.e*. the C—H⋯N hydrogen bonds) in (I), which are barely present in (II). The O⋯H/H⋯O contacts are slightly higher in (II) than (I), presumably reflecting that fact that there are many more C—H⋯O bonds in (II) (compare Table 2 ▸), although the H⋯O contacts are shorter in (I). The percentages of C⋯H/H⋯C contacts in the two compounds are very similar, whereas C⋯C contacts are insignificant in both structures, which presumably correlates with the very weak π–π stacking described above. Finally, S⋯H/H⋯S contacts are clearly more prominent in (I) although any C—H⋯S bonds in (I) would be regarded as very weak at best (shortest H⋯S separation = 2.95 Å). When the two mol­ecules in the asymmetric unit of (I) are compared with each other (Table 3 ▸), there is little difference between them and the same applies to (II).

## Database survey   

A survey of the Cambridge Structural Database (Groom *et al.*, 2016 ▸) updated to September 2017 for the common central –CH=N—N(CH_3_)—C(=O)—CH_2_– fragment of the title compounds revealed seven matches, *viz*. ALAHEC (Cardoso *et al.*, 2016*b* ▸); FOTMUX (Ramírez *et al.*, 2009*a* ▸); KULREP (Ramírez *et al.*, 2009*b* ▸); OFEBIL (Cao *et al.*, 2007 ▸), and EYUBAD, EYUBEH and EYUBIL: this latter trio of refcodes correspond to the three isomeric nitro compounds (Cardoso *et al.*, 2016*a* ▸) noted in the *Chemical Context* section above.

## Synthesis and crystallization   

The appropriate thienyl acetohydrazide derivative (Cardoso *et al.*, 2014 ▸) (0.20 g, 1.0 equiv.) was suspended in acetone (5 ml) and potassium carbonate (4.0 equiv.) was added. The reaction mixture was stirred at room temperature for 30 minutes and methyl iodide (4.0 equiv.) was added. The reaction mixture was maintained at 313 K, until TLC indicated the reaction was complete. The mixture was then rotary evaporated to leave a residue, which was dissolved in water (20 ml) and extracted with ethyl acetate (3 × 10 ml). The organic fractions were combined, dried with anhydrous MgSO_4_, filtered and the solvent evaporated at reduced pressure. The crystals used for the intensity data collections were recrystallized from methanol solution at room temperature to yield colourless plates of (I) and colourless slabs of (II).

(*E*)-*N*′-(3-Cyano­benzyl­idene)-*N*-methyl-2-(thio­phen-2-yl)acetohydrazide, (I). Yield: 78%; yellow solid; m.p. 690–692 K. ^1^H NMR (400 MHz; DMSO) δ: 8.24 (1H; *s*; N=CH), 8.14 (1H; *d*; *J*_HH_ = 7.9 Hz; H-11′), 8.04 (1H; *s*; H-7′), 7.87 (1H; *d*; *J*_HH_ = 7.7 Hz; H-9′), 7.70–7.66 (1H; *m*; H-10′), 7.36 (1H; *dd*; *J*_HH_ = 5.1 and 1.2 Hz; H-5), 6.99–6.98 (1H; *m*; H-3), 6.96–6.94 (1H; *m*; H-4) 4.41 (2H; *s*; CH_2_), 3.33 (3H; *s*; N-CH_3_). ^13^C NMR (125 MHz; DMSO) δ: 170.9 (C=O), 138.5 (N=CH), 137.0 (C-2), 136.0 (C-6′ and C-9′), 132.8 (C-11′), 131.4 (C-7′), 130.4 (C-10′), 130.0 (C-3), 126.7 (C-4), 125.2 (C-5), 118.5 (CN), 111.9 (C-8′), 34.3 (N—CH_3_), 28.1 (CH_2_). MS/ESI: [*M* + Na]: 306. IR ν_max_ (cm^−1^; KBr pellets): 1678 (C=O); 3101 (N—CH_3_).

(*E*)-*N*′-(4-Meth­oxy­benzyl­idene)-*N*-methyl-2-(thio­phen-2-yl)acetohydrazide, (II). Yield: 62%; yellow solid; m.p. 629–630 K. ^1^H NMR (400 MHz; DMSO) δ: 7.94 (1H; *s*; N=CH), 7.76 (2H; *d*; *J*_HH_ = 8.6; H-7′ and H-11′), 7.34 (1H; *d*; *J*_HH_ = 4.8 Hz; H-5), 7.03 (2H; *d*; *J*_HH_ = 8.6 Hz; H-9′ and H-8′ and H-10′), 6.97–6.93 (2H; *m*; H-3 and H-4), 4.34 (2H; *s*; CH_2_), 3.81 (3H; *s*; OCH_3_) 3.33 (3H; *s*; N-CH_3_). ^13^C NMR (125 MHz; DMSO) δ: 170.4 (C=O), 160.5 (C-9′), 140.4 (N=CH), 137.2 (C-2), 128.6 (C-7′ and C-11′), 127.3 (C-3), 126.5 (C-6′), 126.4 (C-4), 125.0 (C-5), 114.2 (C-8′ and C-10′), 55.2 (OCH_3_), 34.3 (N—CH_3_), 27.8 (CH_2_). MS/ESI: [*M* + Na]: 299. IR ν_max_ (cm^−1^; KBr pellet): 1668 (C=O); 2962 (N—CH_3_).

## Refinement   

Crystal data, data collection and structure refinement details are summarized in Table 4 ▸. The hydrogen atoms were geometrically placed (C—H = 0.95–1.00 Å) and refined as riding atoms. The constraint *U*_iso_(H) = 1.2*U*_eq_(carrier) or 1.5*U*_eq_(methyl carrier) was applied in all cases. The N-methyl group was allowed to rotate, but not to tip, to best fit the electron density (AFIX 137 instruction): in every case this group rotated from its initial orientation to minimize steric inter­action with H7; the final orientation leads to a rather short C8—H⋯O1 intra­molecular contact but we do not regard this as a bond. The thio­phene rings in both mol­ecules of (II) show ‘flip’ rotational disorder.

## Supplementary Material

Crystal structure: contains datablock(s) I, II, global. DOI: 10.1107/S2056989017014384/vm2205sup1.cif

Structure factors: contains datablock(s) I. DOI: 10.1107/S2056989017014384/vm2205Isup2.hkl

Structure factors: contains datablock(s) II. DOI: 10.1107/S2056989017014384/vm2205IIsup3.hkl

CCDC references: 1578318, 1578317

Additional supporting information:  crystallographic information; 3D view; checkCIF report

## Figures and Tables

**Table 1:** Hydrogen-bond geometry (Å, °) for (I) *Cg*1, *Cg*2, *Cg*3 and *Cg*4 are the centroids of the C11–C14/S1, C1–C6, C26–C29/S2 and C16–C21 rings, respectively.

*D*—H⋯*A*	*D*—H	H⋯*A*	*D*⋯*A*	*D*—H⋯*A*
C4—H4⋯O1^i^	0.95	2.31	3.1691 (19)	151
C7—H7⋯N6^ii^	0.95	2.53	3.452 (2)	164
C19—H19⋯O2^i^	0.95	2.27	3.1980 (19)	164
C22—H22⋯N3^iii^	0.95	2.61	3.536 (2)	164
C10—H10*B*⋯*Cg*4^iv^	0.99	2.97	3.4724 (18)	113
C12—H12⋯*Cg*3	0.95	2.60	3.436 (2)	147
C23—H23*C*⋯*Cg*2^iv^	0.98	2.90	3.5646 (19)	126
C25—H25*B*⋯*Cg*1^v^	0.99	2.71	3.6910 (18)	169

Symmetry codes: (i) 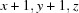; (ii) 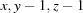; (iii) 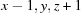; (iv) 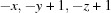; (v) 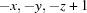.

**Table 2:** Hydrogen-bond geometry (Å, °) for (II) *Cg*1, *Cg*2, *Cg*3 and *Cg*4 are the centroids of the C11–C14/S1, C1–C6, C26–C29/S2 and C16–C21 rings, respectively.

*D*—H⋯*A*	*D*—H	H⋯*A*	*D*⋯*A*	*D*—H⋯*A*
C6—H6⋯S1^i^	0.95	2.87	3.7326 (14)	152
C10—H10*B*⋯O3	0.99	2.56	3.5366 (19)	169
C13—H13⋯O2^ii^	0.95	2.58	3.499 (2)	164
C15—H15*A*⋯O3^iii^	0.98	2.50	3.478 (2)	176
C25—H25*A*⋯O1	0.99	2.38	3.3206 (19)	157
C28—H28⋯O4^iv^	0.95	2.42	3.307 (2)	156
C29—H29⋯O1^v^	0.95	2.50	3.422 (2)	163
C30—H30*A*⋯O1^vi^	0.98	2.45	3.419 (2)	168
C6—H6⋯*Cg*1^i^	0.95	2.67	3.6071 (15)	169
C8—H8*C*⋯*Cg*2^i^	0.98	2.72	3.4831 (16)	135
C21—H21⋯*Cg*3^vii^	0.95	2.90	3.6721 (18)	140
C23—H23*A*⋯*Cg*4^vii^	0.98	2.81	3.6560 (16)	145
C23—H23*C*⋯*Cg*4^viii^	0.98	2.88	3.6067 (16)	131

Symmetry codes: (i) 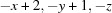; (ii) 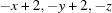; (iii) 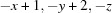; (iv) 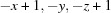; (v) 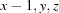; (vi) 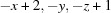; (vii) 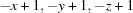; (viii) 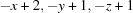.

**Table 3:** Hirshfeld contact inter­actions (%)

Contact type	(I) *A*	(I) *B*	(I)	(II) *A*	(II) *B*	(II)
H⋯H	30.8	35.1	33.0	51.0	53.9	52.5
C⋯H/H⋯C	27.1	25.7	26.4	23.9	22.6	23.2
O⋯H/H⋯O	8.4	9.4	8.9	13.7	14.5	14.1
N⋯H/H⋯N	16.2	14.1	15.1	2.6	2.3	2.4
C⋯C	2.9	3.0	2.9	2.1	1.6	1.8
C⋯N/N⋯C	2.8	3.8	3.3	1.8	2.6	2.2
S⋯H/H⋯S	9.0	6.9	7.9	3.5	2.2	2.9
others	2.8	2.0	2.4	1.4	0.3	0.9

**Table 4:** Experimental details

	(I)	(II)
Crystal data		
Chemical formula	C_15_H_13_N_3_OS	C_15_H_16_N_2_O_2_S
*M*_r_	283.34	288.36
Crystal system, space group	Triclinic, *P* 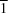	Triclinic, *P* 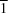
Temperature (K)	100	100
*a*, *b*, *c* (Å)	9.3594 (7), 10.1143 (7), 15.8070 (12)	7.2148 (5), 8.8307 (5), 24.1120 (17)
α, β, γ (°)	106.704 (5), 92.432 (7), 106.777 (5)	80.025 (6), 87.601 (7), 76.097 (6)
*V* (Å^3^)	1359.31 (18)	1468.67 (17)
*Z*	4	4
Radiation type	Mo *K*α	Mo *K*α
μ (mm^−1^)	0.24	0.22
Crystal size (mm)	0.18 × 0.12 × 0.03	0.19 × 0.13 × 0.05

Data collection		
Diffractometer	Rigaku Mercury CCD	Rigaku Mercury CCD
Absorption correction	Multi-scan (*CrystalClear*; Rigaku, 2012 ▸)	Multi-scan (*CrystalClear*; Rigaku, 2012 ▸)
*T*_min_, *T*_max_	0.786, 1.000	0.820, 1.000
No. of measured, independent and observed [*I* > 2σ(*I*)] reflections	18105, 6217, 5204	19273, 6732, 5764
*R*_int_	0.034	0.038
(sin θ/λ)_max_ (Å^−1^)	0.650	0.649

Refinement		
*R*[*F* ^2^ > 2σ(*F* ^2^)], *wR*(*F* ^2^), *S*	0.038, 0.104, 1.04	0.044, 0.127, 1.06
No. of reflections	6217	6732
No. of parameters	363	367
H-atom treatment	H-atom parameters constrained	H-atom parameters constrained
Δρ_max_, Δρ_min_ (e Å^−3^)	0.51, −0.32	0.63, −0.51

Computer programs: *CrystalClear* (Rigaku, 2012 ▸), *SHELXS97* (Sheldrick, 2008 ▸), *SHELXL2014* (Sheldrick, 2015 ▸), *ORTEP-3 for Windows* (Farrugia, 2012 ▸) and *publCIF* (Westrip, 2010 ▸).

## supplementary crystallographic information

### (E)-N'-(3-Cyanobenzylidene)-N-methyl-2-(thiophen-2-yl)acetohydrazide (I) Crystal data

**Table:**

C_15_H_13_N_3_OS	*Z* = 4
*M**_r_* = 283.34	*F*(000) = 592
Triclinic, *P*1	*D*_x_ = 1.385 Mg m^−^^3^
*a* = 9.3594 (7) Å	Mo *K*α radiation, λ = 0.71075 Å
*b* = 10.1143 (7) Å	Cell parameters from 15155 reflections
*c* = 15.8070 (12) Å	θ = 2.2–27.5°
α = 106.704 (5)°	µ = 0.24 mm^−^^1^
β = 92.432 (7)°	*T* = 100 K
γ = 106.777 (5)°	Plate, colourless
*V* = 1359.31 (18) Å^3^	0.18 × 0.12 × 0.03 mm

### (E)-N'-(3-Cyanobenzylidene)-N-methyl-2-(thiophen-2-yl)acetohydrazide (I) Data collection

**Table:**

Rigaku Mercury CCD diffractometer	5204 reflections with *I* > 2σ(*I*)
ω scans	*R*_int_ = 0.034
Absorption correction: multi-scan (CrystalClear; Rigaku, 2012)	θ_max_ = 27.5°, θ_min_ = 2.6°
*T*_min_ = 0.786, *T*_max_ = 1.000	*h* = −11→12
18105 measured reflections	*k* = −12→13
6217 independent reflections	*l* = −20→20

### (E)-N'-(3-Cyanobenzylidene)-N-methyl-2-(thiophen-2-yl)acetohydrazide (I) Refinement

**Table:**

Refinement on *F*^2^	Primary atom site location: structure-invariant direct methods
Least-squares matrix: full	Hydrogen site location: inferred from neighbouring sites
*R*[*F*^2^ > 2σ(*F*^2^)] = 0.038	H-atom parameters constrained
*wR*(*F*^2^) = 0.104	*w* = 1/[σ^2^(*F*_o_^2^) + (0.0527*P*)^2^ + 0.4808*P*] where *P* = (*F*_o_^2^ + 2*F*_c_^2^)/3
*S* = 1.04	(Δ/σ)_max_ = 0.001
6217 reflections	Δρ_max_ = 0.51 e Å^−^^3^
363 parameters	Δρ_min_ = −0.32 e Å^−^^3^
0 restraints	

### (E)-N'-(3-Cyanobenzylidene)-N-methyl-2-(thiophen-2-yl)acetohydrazide (I) Special details

**Table:**

Geometry. All esds (except the esd in the dihedral angle between two l.s. planes) are estimated using the full covariance matrix. The cell esds are taken into account individually in the estimation of esds in distances, angles and torsion angles; correlations between esds in cell parameters are only used when they are defined by crystal symmetry. An approximate (isotropic) treatment of cell esds is used for estimating esds involving l.s. planes.

### (E)-N'-(3-Cyanobenzylidene)-N-methyl-2-(thiophen-2-yl)acetohydrazide (I) Fractional atomic coordinates and isotropic or equivalent isotropic displacement parameters (Å^2^)

**Table:**

	*x*	*y*	*z*	*U*_iso_*/*U*_eq_	
C1	0.44883 (17)	0.46449 (16)	0.16930 (10)	0.0182 (3)	
C2	0.44634 (18)	0.57507 (16)	0.24628 (10)	0.0198 (3)	
H2	0.3740	0.5550	0.2851	0.024*	
C3	0.54885 (18)	0.71367 (16)	0.26619 (10)	0.0196 (3)	
H3	0.5453	0.7879	0.3182	0.024*	
C4	0.65642 (18)	0.74502 (16)	0.21093 (10)	0.0207 (3)	
H4	0.7269	0.8397	0.2250	0.025*	
C5	0.65933 (17)	0.63477 (16)	0.13419 (10)	0.0183 (3)	
C6	0.55554 (17)	0.49516 (16)	0.11306 (10)	0.0180 (3)	
H6	0.5579	0.4215	0.0604	0.022*	
C7	0.34349 (17)	0.31634 (16)	0.14643 (10)	0.0187 (3)	
H7	0.3492	0.2430	0.0946	0.022*	
C8	0.15716 (19)	0.03219 (16)	0.09561 (10)	0.0227 (3)	
H8A	0.1446	0.0603	0.0421	0.034*	
H8B	0.0780	−0.0588	0.0904	0.034*	
H8C	0.2560	0.0183	0.1018	0.034*	
C9	0.04341 (17)	0.11622 (16)	0.23031 (10)	0.0202 (3)	
C10	0.03819 (17)	0.24178 (16)	0.30984 (10)	0.0196 (3)	
H10A	0.0702	0.3332	0.2944	0.024*	
H10B	−0.0668	0.2255	0.3230	0.024*	
C11	0.13786 (17)	0.25767 (15)	0.39154 (10)	0.0182 (3)	
C12	0.09228 (19)	0.22118 (17)	0.46509 (10)	0.0228 (3)	
H12	−0.0103	0.1799	0.4709	0.027*	
C13	0.2143 (2)	0.25137 (18)	0.53227 (11)	0.0260 (3)	
H13	0.2019	0.2329	0.5876	0.031*	
C14	0.35045 (19)	0.30956 (17)	0.50833 (11)	0.0251 (3)	
H14	0.4440	0.3371	0.5449	0.030*	
C15	0.77123 (18)	0.66406 (16)	0.07614 (10)	0.0201 (3)	
N1	0.24352 (14)	0.28688 (13)	0.19727 (8)	0.0185 (3)	
N2	0.14646 (15)	0.14648 (13)	0.17394 (8)	0.0194 (3)	
N3	0.86018 (16)	0.68439 (14)	0.02951 (9)	0.0244 (3)	
O1	−0.04085 (14)	−0.00766 (12)	0.21693 (8)	0.0268 (3)	
S1	0.33237 (4)	0.32713 (4)	0.40385 (3)	0.02304 (10)	
C16	0.12951 (17)	0.59905 (16)	0.81831 (10)	0.0178 (3)	
C17	0.19830 (17)	0.55084 (16)	0.74397 (10)	0.0193 (3)	
H17	0.1600	0.4524	0.7071	0.023*	
C18	0.32126 (17)	0.64447 (16)	0.72338 (10)	0.0199 (3)	
H18	0.3666	0.6096	0.6726	0.024*	
C19	0.37944 (18)	0.78915 (17)	0.77615 (10)	0.0212 (3)	
H19	0.4638	0.8534	0.7617	0.025*	
C20	0.31126 (18)	0.83843 (16)	0.85109 (10)	0.0204 (3)	
C21	0.18679 (17)	0.74391 (16)	0.87227 (10)	0.0192 (3)	
H21	0.1414	0.7782	0.9232	0.023*	
C22	−0.00144 (17)	0.50111 (16)	0.84135 (10)	0.0183 (3)	
H22	−0.0419	0.5329	0.8948	0.022*	
C23	−0.23822 (18)	0.31465 (17)	0.89282 (10)	0.0212 (3)	
H23A	−0.1578	0.3386	0.9417	0.032*	
H23B	−0.3220	0.2317	0.8951	0.032*	
H23C	−0.2736	0.3987	0.8988	0.032*	
C24	−0.24432 (18)	0.14399 (16)	0.74387 (10)	0.0205 (3)	
C25	−0.17210 (19)	0.11278 (16)	0.65922 (10)	0.0209 (3)	
H25A	−0.0613	0.1532	0.6752	0.025*	
H25B	−0.1977	0.0061	0.6323	0.025*	
C26	−0.21947 (17)	0.17434 (16)	0.59025 (10)	0.0189 (3)	
C27	−0.30906 (18)	0.09509 (18)	0.51024 (11)	0.0240 (3)	
H27	−0.3551	−0.0073	0.4928	0.029*	
C28	−0.32632 (19)	0.18168 (19)	0.45580 (11)	0.0270 (4)	
H28	−0.3847	0.1437	0.3985	0.032*	
C29	−0.24891 (19)	0.32583 (18)	0.49576 (11)	0.0248 (3)	
H29	−0.2465	0.3998	0.4694	0.030*	
C30	0.37254 (18)	0.98796 (17)	0.90659 (11)	0.0225 (3)	
N4	−0.05999 (14)	0.37105 (13)	0.78716 (8)	0.0177 (3)	
N5	−0.18037 (15)	0.27735 (13)	0.80764 (8)	0.0193 (3)	
N6	0.42280 (17)	1.10717 (15)	0.95097 (9)	0.0275 (3)	
O2	−0.35504 (14)	0.05496 (12)	0.75548 (8)	0.0281 (3)	
S2	−0.15679 (5)	0.35686 (4)	0.59911 (3)	0.02189 (10)	

### (E)-N'-(3-Cyanobenzylidene)-N-methyl-2-(thiophen-2-yl)acetohydrazide (I) Atomic displacement parameters (Å^2^)

**Table:**

	*U*^11^	*U*^22^	*U*^33^	*U*^12^	*U*^13^	*U*^23^
C1	0.0199 (8)	0.0169 (7)	0.0193 (7)	0.0065 (6)	0.0007 (6)	0.0074 (6)
C2	0.0208 (8)	0.0197 (7)	0.0198 (7)	0.0069 (6)	0.0033 (6)	0.0069 (6)
C3	0.0223 (8)	0.0176 (7)	0.0174 (7)	0.0065 (6)	0.0021 (6)	0.0029 (6)
C4	0.0212 (8)	0.0175 (7)	0.0223 (8)	0.0042 (6)	0.0014 (6)	0.0068 (6)
C5	0.0192 (8)	0.0195 (7)	0.0179 (7)	0.0068 (6)	0.0022 (6)	0.0080 (6)
C6	0.0201 (8)	0.0179 (7)	0.0171 (7)	0.0080 (6)	0.0015 (6)	0.0055 (6)
C7	0.0207 (8)	0.0165 (7)	0.0175 (7)	0.0060 (6)	−0.0003 (6)	0.0035 (6)
C8	0.0276 (9)	0.0159 (7)	0.0195 (7)	0.0028 (6)	0.0031 (6)	0.0019 (6)
C9	0.0194 (8)	0.0189 (7)	0.0210 (7)	0.0044 (6)	0.0000 (6)	0.0062 (6)
C10	0.0176 (7)	0.0194 (7)	0.0223 (7)	0.0074 (6)	0.0038 (6)	0.0057 (6)
C11	0.0172 (7)	0.0143 (6)	0.0217 (7)	0.0062 (6)	0.0038 (6)	0.0019 (6)
C12	0.0210 (8)	0.0266 (8)	0.0213 (8)	0.0114 (6)	0.0076 (6)	0.0038 (6)
C13	0.0300 (9)	0.0307 (8)	0.0177 (7)	0.0149 (7)	0.0043 (6)	0.0028 (6)
C14	0.0246 (8)	0.0238 (8)	0.0235 (8)	0.0101 (6)	−0.0023 (6)	0.0007 (6)
C15	0.0220 (8)	0.0161 (7)	0.0205 (7)	0.0051 (6)	−0.0003 (6)	0.0046 (6)
N1	0.0191 (7)	0.0142 (6)	0.0205 (6)	0.0035 (5)	−0.0001 (5)	0.0049 (5)
N2	0.0218 (7)	0.0128 (6)	0.0191 (6)	0.0022 (5)	0.0020 (5)	0.0019 (5)
N3	0.0259 (7)	0.0223 (7)	0.0219 (7)	0.0049 (6)	0.0046 (6)	0.0048 (5)
O1	0.0274 (6)	0.0181 (5)	0.0268 (6)	−0.0018 (5)	0.0043 (5)	0.0038 (5)
S1	0.0175 (2)	0.02163 (19)	0.0281 (2)	0.00403 (15)	0.00253 (15)	0.00725 (16)
C16	0.0182 (7)	0.0169 (7)	0.0187 (7)	0.0056 (6)	−0.0002 (6)	0.0065 (6)
C17	0.0202 (8)	0.0169 (7)	0.0201 (7)	0.0059 (6)	0.0003 (6)	0.0052 (6)
C18	0.0206 (8)	0.0225 (7)	0.0174 (7)	0.0080 (6)	0.0033 (6)	0.0063 (6)
C19	0.0197 (8)	0.0216 (7)	0.0224 (8)	0.0042 (6)	0.0005 (6)	0.0095 (6)
C20	0.0226 (8)	0.0170 (7)	0.0201 (7)	0.0046 (6)	−0.0021 (6)	0.0060 (6)
C21	0.0206 (8)	0.0192 (7)	0.0183 (7)	0.0060 (6)	0.0012 (6)	0.0069 (6)
C22	0.0199 (8)	0.0185 (7)	0.0176 (7)	0.0073 (6)	0.0027 (6)	0.0057 (6)
C23	0.0241 (8)	0.0208 (7)	0.0185 (7)	0.0060 (6)	0.0050 (6)	0.0063 (6)
C24	0.0241 (8)	0.0163 (7)	0.0215 (7)	0.0064 (6)	0.0025 (6)	0.0066 (6)
C25	0.0264 (8)	0.0146 (7)	0.0208 (7)	0.0071 (6)	0.0045 (6)	0.0035 (6)
C26	0.0201 (8)	0.0174 (7)	0.0194 (7)	0.0071 (6)	0.0067 (6)	0.0044 (6)
C27	0.0212 (8)	0.0251 (8)	0.0221 (8)	0.0049 (6)	0.0039 (6)	0.0045 (6)
C28	0.0243 (9)	0.0352 (9)	0.0221 (8)	0.0121 (7)	0.0015 (6)	0.0072 (7)
C29	0.0288 (9)	0.0297 (8)	0.0211 (8)	0.0159 (7)	0.0060 (6)	0.0089 (6)
C30	0.0235 (8)	0.0210 (8)	0.0228 (8)	0.0041 (6)	0.0041 (6)	0.0093 (6)
N4	0.0178 (6)	0.0165 (6)	0.0198 (6)	0.0047 (5)	0.0031 (5)	0.0078 (5)
N5	0.0203 (7)	0.0168 (6)	0.0201 (6)	0.0041 (5)	0.0059 (5)	0.0063 (5)
N6	0.0310 (8)	0.0216 (7)	0.0260 (7)	0.0038 (6)	0.0058 (6)	0.0060 (6)
O2	0.0301 (7)	0.0188 (5)	0.0294 (6)	−0.0007 (5)	0.0063 (5)	0.0068 (5)
S2	0.0295 (2)	0.01730 (18)	0.01936 (19)	0.00892 (15)	0.00386 (15)	0.00484 (14)

### (E)-N'-(3-Cyanobenzylidene)-N-methyl-2-(thiophen-2-yl)acetohydrazide (I) Geometric parameters (Å, º)

**Table:**

C1—C6	1.392 (2)	C16—C17	1.396 (2)
C1—C2	1.403 (2)	C16—C21	1.397 (2)
C1—C7	1.467 (2)	C16—C22	1.470 (2)
C2—C3	1.389 (2)	C17—C18	1.381 (2)
C2—H2	0.9500	C17—H17	0.9500
C3—C4	1.388 (2)	C18—C19	1.391 (2)
C3—H3	0.9500	C18—H18	0.9500
C4—C5	1.399 (2)	C19—C20	1.402 (2)
C4—H4	0.9500	C19—H19	0.9500
C5—C6	1.400 (2)	C20—C21	1.399 (2)
C5—C15	1.444 (2)	C20—C30	1.442 (2)
C6—H6	0.9500	C21—H21	0.9500
C7—N1	1.2848 (19)	C22—N4	1.2887 (19)
C7—H7	0.9500	C22—H22	0.9500
C8—N2	1.4607 (19)	C23—N5	1.4615 (19)
C8—H8A	0.9800	C23—H23A	0.9800
C8—H8B	0.9800	C23—H23B	0.9800
C8—H8C	0.9800	C23—H23C	0.9800
C9—O1	1.2246 (18)	C24—O2	1.2210 (19)
C9—N2	1.3702 (19)	C24—N5	1.3766 (19)
C9—C10	1.523 (2)	C24—C25	1.518 (2)
C10—C11	1.502 (2)	C25—C26	1.510 (2)
C10—H10A	0.9900	C25—H25A	0.9900
C10—H10B	0.9900	C25—H25B	0.9900
C11—C12	1.367 (2)	C26—C27	1.373 (2)
C11—S1	1.7316 (16)	C26—S2	1.7294 (15)
C12—C13	1.426 (2)	C27—C28	1.427 (2)
C12—H12	0.9500	C27—H27	0.9500
C13—C14	1.360 (2)	C28—C29	1.368 (2)
C13—H13	0.9500	C28—H28	0.9500
C14—S1	1.7175 (17)	C29—S2	1.7146 (17)
C14—H14	0.9500	C29—H29	0.9500
C15—N3	1.148 (2)	C30—N6	1.151 (2)
N1—N2	1.3797 (17)	N4—N5	1.3651 (17)
			
C6—C1—C2	119.22 (14)	C17—C16—C21	119.18 (14)
C6—C1—C7	118.67 (13)	C17—C16—C22	121.66 (14)
C2—C1—C7	122.10 (14)	C21—C16—C22	119.17 (13)
C3—C2—C1	120.46 (14)	C18—C17—C16	120.83 (14)
C3—C2—H2	119.8	C18—C17—H17	119.6
C1—C2—H2	119.8	C16—C17—H17	119.6
C4—C3—C2	120.75 (14)	C17—C18—C19	120.78 (14)
C4—C3—H3	119.6	C17—C18—H18	119.6
C2—C3—H3	119.6	C19—C18—H18	119.6
C3—C4—C5	118.88 (14)	C18—C19—C20	118.77 (14)
C3—C4—H4	120.6	C18—C19—H19	120.6
C5—C4—H4	120.6	C20—C19—H19	120.6
C4—C5—C6	120.80 (14)	C21—C20—C19	120.65 (14)
C4—C5—C15	119.99 (13)	C21—C20—C30	120.45 (14)
C6—C5—C15	119.21 (13)	C19—C20—C30	118.90 (14)
C1—C6—C5	119.88 (14)	C16—C21—C20	119.79 (14)
C1—C6—H6	120.1	C16—C21—H21	120.1
C5—C6—H6	120.1	C20—C21—H21	120.1
N1—C7—C1	119.32 (13)	N4—C22—C16	118.31 (13)
N1—C7—H7	120.3	N4—C22—H22	120.8
C1—C7—H7	120.3	C16—C22—H22	120.8
N2—C8—H8A	109.5	N5—C23—H23A	109.5
N2—C8—H8B	109.5	N5—C23—H23B	109.5
H8A—C8—H8B	109.5	H23A—C23—H23B	109.5
N2—C8—H8C	109.5	N5—C23—H23C	109.5
H8A—C8—H8C	109.5	H23A—C23—H23C	109.5
H8B—C8—H8C	109.5	H23B—C23—H23C	109.5
O1—C9—N2	120.86 (14)	O2—C24—N5	120.83 (14)
O1—C9—C10	121.55 (14)	O2—C24—C25	121.72 (14)
N2—C9—C10	117.60 (13)	N5—C24—C25	117.45 (13)
C11—C10—C9	112.24 (12)	C26—C25—C24	114.41 (13)
C11—C10—H10A	109.2	C26—C25—H25A	108.7
C9—C10—H10A	109.2	C24—C25—H25A	108.7
C11—C10—H10B	109.2	C26—C25—H25B	108.7
C9—C10—H10B	109.2	C24—C25—H25B	108.7
H10A—C10—H10B	107.9	H25A—C25—H25B	107.6
C12—C11—C10	126.61 (14)	C27—C26—C25	125.78 (14)
C12—C11—S1	110.52 (12)	C27—C26—S2	110.37 (12)
C10—C11—S1	122.87 (11)	C25—C26—S2	123.74 (11)
C11—C12—C13	113.18 (15)	C26—C27—C28	113.37 (15)
C11—C12—H12	123.4	C26—C27—H27	123.3
C13—C12—H12	123.4	C28—C27—H27	123.3
C14—C13—C12	112.47 (15)	C29—C28—C27	112.07 (15)
C14—C13—H13	123.8	C29—C28—H28	124.0
C12—C13—H13	123.8	C27—C28—H28	124.0
C13—C14—S1	111.68 (13)	C28—C29—S2	111.79 (12)
C13—C14—H14	124.2	C28—C29—H29	124.1
S1—C14—H14	124.2	S2—C29—H29	124.1
N3—C15—C5	178.57 (16)	N6—C30—C20	179.34 (19)
C7—N1—N2	117.84 (12)	C22—N4—N5	119.44 (13)
C9—N2—N1	116.96 (12)	N4—N5—C24	116.38 (12)
C9—N2—C8	120.85 (12)	N4—N5—C23	122.32 (12)
N1—N2—C8	122.11 (12)	C24—N5—C23	121.30 (13)
C14—S1—C11	92.14 (8)	C29—S2—C26	92.39 (8)
			
C6—C1—C2—C3	0.1 (2)	C21—C16—C17—C18	−0.2 (2)
C7—C1—C2—C3	179.36 (14)	C22—C16—C17—C18	−179.99 (14)
C1—C2—C3—C4	−0.6 (2)	C16—C17—C18—C19	−0.1 (2)
C2—C3—C4—C5	0.5 (2)	C17—C18—C19—C20	0.3 (2)
C3—C4—C5—C6	0.2 (2)	C18—C19—C20—C21	−0.2 (2)
C3—C4—C5—C15	−179.27 (14)	C18—C19—C20—C30	179.21 (14)
C2—C1—C6—C5	0.5 (2)	C17—C16—C21—C20	0.2 (2)
C7—C1—C6—C5	−178.73 (13)	C22—C16—C21—C20	−179.92 (14)
C4—C5—C6—C1	−0.7 (2)	C19—C20—C21—C16	−0.1 (2)
C15—C5—C6—C1	178.75 (13)	C30—C20—C21—C16	−179.44 (14)
C6—C1—C7—N1	−179.33 (14)	C17—C16—C22—N4	−5.5 (2)
C2—C1—C7—N1	1.4 (2)	C21—C16—C22—N4	174.70 (14)
O1—C9—C10—C11	87.85 (18)	O2—C24—C25—C26	97.62 (18)
N2—C9—C10—C11	−91.98 (16)	N5—C24—C25—C26	−82.29 (18)
C9—C10—C11—C12	−106.11 (17)	C24—C25—C26—C27	−109.77 (17)
C9—C10—C11—S1	73.77 (15)	C24—C25—C26—S2	74.51 (17)
C10—C11—C12—C13	−179.17 (14)	C25—C26—C27—C28	−175.81 (14)
S1—C11—C12—C13	0.93 (17)	S2—C26—C27—C28	0.39 (18)
C11—C12—C13—C14	−0.3 (2)	C26—C27—C28—C29	0.0 (2)
C12—C13—C14—S1	−0.46 (18)	C27—C28—C29—S2	−0.47 (18)
C1—C7—N1—N2	−179.87 (12)	C16—C22—N4—N5	179.09 (13)
O1—C9—N2—N1	−175.82 (14)	C22—N4—N5—C24	175.30 (13)
C10—C9—N2—N1	4.0 (2)	C22—N4—N5—C23	−5.4 (2)
O1—C9—N2—C8	0.9 (2)	O2—C24—N5—N4	−177.92 (14)
C10—C9—N2—C8	−179.28 (13)	C25—C24—N5—N4	2.0 (2)
C7—N1—N2—C9	178.23 (14)	O2—C24—N5—C23	2.7 (2)
C7—N1—N2—C8	1.6 (2)	C25—C24—N5—C23	−177.35 (13)
C13—C14—S1—C11	0.84 (13)	C28—C29—S2—C26	0.59 (13)
C12—C11—S1—C14	−1.01 (12)	C27—C26—S2—C29	−0.55 (12)
C10—C11—S1—C14	179.09 (12)	C25—C26—S2—C29	175.74 (13)

### (E)-N'-(3-Cyanobenzylidene)-N-methyl-2-(thiophen-2-yl)acetohydrazide (I) Hydrogen-bond geometry (Å, º)

Cg1, Cg2, Cg3 and Cg4 are the centroids of the C11–C14/S1, C1–C6, C26–C29/S2 and C16–C21 rings, respectively.

**Table:**

*D*—H···*A*	*D*—H	H···*A*	*D*···*A*	*D*—H···*A*
C4—H4···O1^i^	0.95	2.31	3.1691 (19)	151
C7—H7···N6^ii^	0.95	2.53	3.452 (2)	164
C19—H19···O2^i^	0.95	2.27	3.1980 (19)	164
C22—H22···N3^iii^	0.95	2.61	3.536 (2)	164
C10—H10*B*···*Cg*4^iv^	0.99	2.97	3.4724 (18)	113
C12—H12···*Cg*3	0.95	2.60	3.436 (2)	147
C23—H23*C*···*Cg*2^iv^	0.98	2.90	3.5646 (19)	126
C25—H25*B*···*Cg*1^v^	0.99	2.71	3.6910 (18)	169

Symmetry codes: (i) *x*+1, *y*+1, *z*; (ii) *x*, *y*−1, *z*−1; (iii) *x*−1, *y*, *z*+1; (iv) −*x*, −*y*+1, −*z*+1; (v) −*x*, −*y*, −*z*+1.

### (E)-N'-(4-Methoxybenzylidene)-N-methyl-2-(thiophen-2-yl)acetohydrazide (II) Crystal data

**Table:**

C_15_H_16_N_2_O_2_S	*Z* = 4
*M**_r_* = 288.36	*F*(000) = 608
Triclinic, *P*1	*D*_x_ = 1.304 Mg m^−^^3^
*a* = 7.2148 (5) Å	Mo *K*α radiation, λ = 0.71073 Å
*b* = 8.8307 (5) Å	Cell parameters from 16502 reflections
*c* = 24.1120 (17) Å	θ = 2.4–27.5°
α = 80.025 (6)°	µ = 0.22 mm^−^^1^
β = 87.601 (7)°	*T* = 100 K
γ = 76.097 (6)°	Slab, colourless
*V* = 1468.67 (17) Å^3^	0.19 × 0.13 × 0.05 mm

### (E)-N'-(4-Methoxybenzylidene)-N-methyl-2-(thiophen-2-yl)acetohydrazide (II) Data collection

**Table:**

Rigaku Mercury CCD diffractometer	5764 reflections with *I* > 2σ(*I*)
ω scans	*R*_int_ = 0.038
Absorption correction: multi-scan (CrystalClear; Rigaku, 2012)	θ_max_ = 27.5°, θ_min_ = 2.4°
*T*_min_ = 0.820, *T*_max_ = 1.000	*h* = −9→9
19273 measured reflections	*k* = −11→11
6732 independent reflections	*l* = −31→30

### (E)-N'-(4-Methoxybenzylidene)-N-methyl-2-(thiophen-2-yl)acetohydrazide (II) Refinement

**Table:**

Refinement on *F*^2^	Primary atom site location: structure-invariant direct methods
Least-squares matrix: full	Hydrogen site location: inferred from neighbouring sites
*R*[*F*^2^ > 2σ(*F*^2^)] = 0.044	H-atom parameters constrained
*wR*(*F*^2^) = 0.127	*w* = 1/[σ^2^(*F*_o_^2^) + (0.0663*P*)^2^ + 0.5738*P*] where *P* = (*F*_o_^2^ + 2*F*_c_^2^)/3
*S* = 1.06	(Δ/σ)_max_ < 0.001
6732 reflections	Δρ_max_ = 0.63 e Å^−^^3^
367 parameters	Δρ_min_ = −0.51 e Å^−^^3^
0 restraints	

### (E)-N'-(4-Methoxybenzylidene)-N-methyl-2-(thiophen-2-yl)acetohydrazide (II) Special details

**Table:**

Geometry. All esds (except the esd in the dihedral angle between two l.s. planes) are estimated using the full covariance matrix. The cell esds are taken into account individually in the estimation of esds in distances, angles and torsion angles; correlations between esds in cell parameters are only used when they are defined by crystal symmetry. An approximate (isotropic) treatment of cell esds is used for estimating esds involving l.s. planes.

### (E)-N'-(4-Methoxybenzylidene)-N-methyl-2-(thiophen-2-yl)acetohydrazide (II) Fractional atomic coordinates and isotropic or equivalent isotropic displacement parameters (Å^2^)

**Table:**

	*x*	*y*	*z*	*U*_iso_*/*U*_eq_	Occ. (<1)
C1	0.67008 (18)	0.60106 (16)	−0.02475 (6)	0.0180 (3)	
C2	0.5794 (2)	0.75469 (16)	−0.01600 (6)	0.0203 (3)	
H2	0.5751	0.7804	0.0208	0.024*	
C3	0.4965 (2)	0.86847 (16)	−0.06029 (6)	0.0218 (3)	
H3	0.4357	0.9720	−0.0539	0.026*	
C4	0.50131 (19)	0.83212 (16)	−0.11471 (6)	0.0200 (3)	
C5	0.5850 (2)	0.67911 (16)	−0.12395 (6)	0.0203 (3)	
H5	0.5857	0.6529	−0.1606	0.024*	
C6	0.66768 (19)	0.56475 (16)	−0.07866 (6)	0.0194 (3)	
H6	0.7235	0.4600	−0.0847	0.023*	
C7	0.76607 (19)	0.48091 (16)	0.02163 (6)	0.0187 (3)	
H7	0.8145	0.3742	0.0166	0.022*	
C8	0.9558 (2)	0.24722 (16)	0.10720 (6)	0.0214 (3)	
H8A	0.8552	0.1999	0.0968	0.032*	
H8B	1.0132	0.1882	0.1430	0.032*	
H8C	1.0541	0.2431	0.0778	0.032*	
C9	0.8960 (2)	0.46599 (17)	0.16159 (6)	0.0214 (3)	
C10	0.8168 (2)	0.64228 (17)	0.16296 (6)	0.0235 (3)	
H10A	0.6984	0.6816	0.1403	0.028*	
H10B	0.7852	0.6583	0.2022	0.028*	
C11	0.9622 (2)	0.73367 (16)	0.13961 (6)	0.0208 (3)	
C12	1.13089 (14)	0.74915 (10)	0.17678 (4)	0.0390 (3)	0.662 (2)
H12	1.1602	0.7098	0.2154	0.047*	0.662 (2)
S1A	1.13089 (14)	0.74915 (10)	0.17678 (4)	0.0390 (3)	0.338 (2)
C13	1.2333 (2)	0.84657 (19)	0.13194 (8)	0.0360 (4)	
H13	1.3437	0.8787	0.1407	0.043*	
C14	1.1583 (2)	0.88417 (18)	0.07941 (8)	0.0320 (4)	
H14	1.2118	0.9443	0.0492	0.038*	
C15	0.4195 (3)	0.9248 (2)	−0.21167 (7)	0.0349 (4)	
H15A	0.3625	1.0230	−0.2370	0.052*	
H15B	0.3453	0.8461	−0.2132	0.052*	
H15C	0.5512	0.8841	−0.2234	0.052*	
N1	0.78323 (16)	0.52285 (13)	0.06906 (5)	0.0181 (2)	
N2	0.87351 (17)	0.41187 (13)	0.11301 (5)	0.0188 (2)	
O1	0.98112 (17)	0.37640 (13)	0.20239 (4)	0.0287 (2)	
O2	0.41861 (15)	0.95573 (12)	−0.15535 (4)	0.0257 (2)	
S1	0.96968 (7)	0.81888 (6)	0.07181 (2)	0.02516 (17)	0.662 (2)
C12A	0.96968 (7)	0.81888 (6)	0.07181 (2)	0.02516 (17)	0.338 (2)
H12A	0.8915	0.8263	0.0402	0.030*	0.338 (2)
C16	0.7764 (2)	0.37271 (18)	0.53247 (6)	0.0232 (3)	
C17	0.8223 (2)	0.21881 (18)	0.51957 (6)	0.0259 (3)	
H17	0.8225	0.2045	0.4814	0.031*	
C18	0.8670 (2)	0.08863 (18)	0.56149 (7)	0.0273 (3)	
H18	0.8965	−0.0147	0.5522	0.033*	
C19	0.8691 (2)	0.10806 (18)	0.61799 (6)	0.0246 (3)	
C20	0.8182 (2)	0.25909 (19)	0.63179 (6)	0.0263 (3)	
H20	0.8150	0.2731	0.6700	0.032*	
C21	0.7718 (2)	0.38993 (18)	0.58890 (7)	0.0261 (3)	
H21	0.7363	0.4931	0.5984	0.031*	
C22	0.7391 (2)	0.51354 (18)	0.48843 (6)	0.0246 (3)	
H22	0.6957	0.6158	0.4983	0.030*	
C23	0.6759 (2)	0.79073 (19)	0.40919 (7)	0.0303 (3)	
H23A	0.5514	0.8036	0.4280	0.045*	
H23B	0.6659	0.8684	0.3744	0.045*	
H23C	0.7702	0.8073	0.4342	0.045*	
C24	0.7715 (2)	0.60876 (19)	0.34096 (7)	0.0273 (3)	
C25	0.8229 (2)	0.43990 (19)	0.32963 (6)	0.0269 (3)	
H25A	0.8984	0.4346	0.2945	0.032*	
H25B	0.9018	0.3700	0.3609	0.032*	
C26	0.6444 (2)	0.38433 (19)	0.32416 (7)	0.0270 (3)	
C27	0.58683 (12)	0.23749 (10)	0.36718 (4)	0.0472 (3)	0.549 (3)
H27	0.6492	0.1698	0.3994	0.057*	0.549 (3)
S2A	0.58683 (12)	0.23749 (10)	0.36718 (4)	0.0472 (3)	0.451 (3)
C28	0.3942 (3)	0.2460 (3)	0.33734 (10)	0.0525 (6)	
H28	0.3185	0.1725	0.3502	0.063*	
C29	0.3408 (3)	0.3617 (3)	0.29275 (9)	0.0481 (5)	
H29	0.2236	0.3762	0.2737	0.058*	
C30	0.9352 (3)	−0.0128 (2)	0.71366 (7)	0.0395 (4)	
H30A	0.9796	−0.1178	0.7365	0.059*	
H30B	0.8089	0.0380	0.7268	0.059*	
H30C	1.0253	0.0522	0.7172	0.059*	
N3	0.76545 (17)	0.49815 (15)	0.43654 (5)	0.0236 (3)	
N4	0.73577 (18)	0.63152 (15)	0.39574 (5)	0.0254 (3)	
O3	0.7577 (2)	0.72180 (15)	0.30224 (5)	0.0378 (3)	
O4	0.92322 (17)	−0.02841 (13)	0.65599 (5)	0.0317 (3)	
S2	0.48492 (10)	0.47394 (10)	0.27350 (3)	0.0423 (3)	0.549 (3)
C27A	0.48492 (10)	0.47394 (10)	0.27350 (3)	0.0423 (3)	0.451 (3)
H27A	0.4809	0.5607	0.2438	0.051*	0.451 (3)

### (E)-N'-(4-Methoxybenzylidene)-N-methyl-2-(thiophen-2-yl)acetohydrazide (II) Atomic displacement parameters (Å^2^)

**Table:**

	*U*^11^	*U*^22^	*U*^33^	*U*^12^	*U*^13^	*U*^23^
C1	0.0141 (6)	0.0180 (6)	0.0235 (7)	−0.0069 (5)	0.0000 (5)	−0.0033 (5)
C2	0.0184 (6)	0.0208 (6)	0.0234 (7)	−0.0052 (5)	−0.0003 (5)	−0.0073 (5)
C3	0.0185 (6)	0.0174 (6)	0.0298 (8)	−0.0027 (5)	−0.0019 (5)	−0.0061 (5)
C4	0.0149 (6)	0.0189 (6)	0.0262 (7)	−0.0057 (5)	−0.0022 (5)	−0.0006 (5)
C5	0.0189 (6)	0.0221 (7)	0.0215 (7)	−0.0071 (5)	0.0001 (5)	−0.0045 (5)
C6	0.0176 (6)	0.0170 (6)	0.0247 (7)	−0.0054 (5)	0.0012 (5)	−0.0046 (5)
C7	0.0171 (6)	0.0163 (6)	0.0239 (7)	−0.0064 (5)	0.0015 (5)	−0.0037 (5)
C8	0.0213 (7)	0.0157 (6)	0.0276 (7)	−0.0059 (5)	−0.0013 (5)	−0.0022 (5)
C9	0.0230 (7)	0.0221 (7)	0.0195 (7)	−0.0085 (5)	0.0030 (5)	−0.0013 (5)
C10	0.0276 (7)	0.0225 (7)	0.0210 (7)	−0.0068 (6)	0.0047 (5)	−0.0053 (5)
C11	0.0238 (7)	0.0161 (6)	0.0224 (7)	−0.0031 (5)	0.0001 (5)	−0.0047 (5)
C12	0.0445 (6)	0.0307 (5)	0.0468 (6)	−0.0145 (4)	0.0042 (4)	−0.0129 (4)
S1A	0.0445 (6)	0.0307 (5)	0.0468 (6)	−0.0145 (4)	0.0042 (4)	−0.0129 (4)
C13	0.0294 (8)	0.0229 (7)	0.0597 (12)	−0.0078 (6)	−0.0071 (8)	−0.0141 (7)
C14	0.0331 (8)	0.0169 (7)	0.0420 (9)	−0.0014 (6)	0.0092 (7)	−0.0026 (6)
C15	0.0456 (10)	0.0298 (8)	0.0263 (8)	−0.0068 (7)	−0.0088 (7)	0.0022 (6)
N1	0.0154 (5)	0.0176 (5)	0.0213 (6)	−0.0051 (4)	−0.0005 (4)	−0.0012 (4)
N2	0.0196 (6)	0.0159 (5)	0.0209 (6)	−0.0053 (4)	−0.0010 (4)	−0.0010 (4)
O1	0.0384 (6)	0.0264 (5)	0.0201 (5)	−0.0084 (5)	−0.0030 (4)	0.0015 (4)
O2	0.0263 (5)	0.0213 (5)	0.0265 (5)	−0.0029 (4)	−0.0059 (4)	0.0011 (4)
S1	0.0214 (3)	0.0195 (2)	0.0325 (3)	−0.00284 (18)	0.00245 (19)	−0.00200 (19)
C12A	0.0214 (3)	0.0195 (2)	0.0325 (3)	−0.00284 (18)	0.00245 (19)	−0.00200 (19)
C16	0.0202 (7)	0.0271 (7)	0.0231 (7)	−0.0073 (5)	−0.0001 (5)	−0.0036 (6)
C17	0.0260 (7)	0.0289 (7)	0.0232 (7)	−0.0057 (6)	0.0010 (6)	−0.0066 (6)
C18	0.0273 (8)	0.0247 (7)	0.0306 (8)	−0.0051 (6)	0.0006 (6)	−0.0079 (6)
C19	0.0205 (7)	0.0257 (7)	0.0269 (8)	−0.0072 (5)	−0.0023 (5)	0.0008 (6)
C20	0.0280 (8)	0.0305 (8)	0.0219 (7)	−0.0093 (6)	−0.0012 (6)	−0.0045 (6)
C21	0.0279 (8)	0.0238 (7)	0.0276 (8)	−0.0067 (6)	−0.0010 (6)	−0.0059 (6)
C22	0.0233 (7)	0.0249 (7)	0.0262 (7)	−0.0071 (6)	−0.0015 (6)	−0.0033 (6)
C23	0.0324 (8)	0.0263 (7)	0.0330 (8)	−0.0101 (6)	−0.0020 (6)	−0.0025 (6)
C24	0.0249 (7)	0.0348 (8)	0.0243 (7)	−0.0137 (6)	−0.0049 (6)	−0.0004 (6)
C25	0.0237 (7)	0.0349 (8)	0.0224 (7)	−0.0088 (6)	−0.0016 (6)	−0.0024 (6)
C26	0.0248 (7)	0.0327 (8)	0.0258 (7)	−0.0075 (6)	−0.0005 (6)	−0.0101 (6)
C27	0.0372 (5)	0.0359 (4)	0.0734 (7)	−0.0118 (3)	−0.0040 (4)	−0.0176 (4)
S2A	0.0372 (5)	0.0359 (4)	0.0734 (7)	−0.0118 (3)	−0.0040 (4)	−0.0176 (4)
C28	0.0597 (13)	0.0571 (13)	0.0596 (13)	−0.0358 (11)	0.0136 (11)	−0.0336 (11)
C29	0.0273 (9)	0.0755 (15)	0.0493 (12)	−0.0116 (9)	0.0014 (8)	−0.0334 (11)
C30	0.0535 (11)	0.0371 (9)	0.0285 (9)	−0.0192 (8)	−0.0129 (8)	0.0075 (7)
N3	0.0208 (6)	0.0265 (6)	0.0237 (6)	−0.0088 (5)	−0.0018 (5)	−0.0002 (5)
N4	0.0261 (6)	0.0255 (6)	0.0255 (6)	−0.0102 (5)	−0.0030 (5)	−0.0001 (5)
O3	0.0526 (8)	0.0360 (6)	0.0263 (6)	−0.0197 (6)	−0.0046 (5)	0.0047 (5)
O4	0.0369 (6)	0.0264 (6)	0.0304 (6)	−0.0091 (5)	−0.0075 (5)	0.0030 (4)
S2	0.0266 (4)	0.0548 (5)	0.0487 (5)	−0.0044 (3)	−0.0049 (3)	−0.0236 (3)
C27A	0.0266 (4)	0.0548 (5)	0.0487 (5)	−0.0044 (3)	−0.0049 (3)	−0.0236 (3)

### (E)-N'-(4-Methoxybenzylidene)-N-methyl-2-(thiophen-2-yl)acetohydrazide (II) Geometric parameters (Å, º)

**Table:**

C1—C6	1.3935 (19)	C16—C21	1.393 (2)
C1—C2	1.4051 (18)	C16—C17	1.405 (2)
C1—C7	1.4686 (19)	C16—C22	1.467 (2)
C2—C3	1.379 (2)	C17—C18	1.376 (2)
C2—H2	0.9500	C17—H17	0.9500
C3—C4	1.401 (2)	C18—C19	1.403 (2)
C3—H3	0.9500	C18—H18	0.9500
C4—O2	1.3687 (17)	C19—O4	1.3659 (18)
C4—C5	1.3931 (19)	C19—C20	1.390 (2)
C5—C6	1.3970 (19)	C20—C21	1.395 (2)
C5—H5	0.9500	C20—H20	0.9500
C6—H6	0.9500	C21—H21	0.9500
C7—N1	1.2811 (18)	C22—N3	1.283 (2)
C7—H7	0.9500	C22—H22	0.9500
C8—N2	1.4611 (17)	C23—N4	1.457 (2)
C8—H8A	0.9800	C23—H23A	0.9800
C8—H8B	0.9800	C23—H23B	0.9800
C8—H8C	0.9800	C23—H23C	0.9800
C9—O1	1.2322 (18)	C24—O3	1.231 (2)
C9—N2	1.3690 (18)	C24—N4	1.375 (2)
C9—C10	1.5297 (19)	C24—C25	1.517 (2)
C10—C11	1.506 (2)	C25—C26	1.502 (2)
C10—H10A	0.9900	C25—H25A	0.9900
C10—H10B	0.9900	C25—H25B	0.9900
C11—S1A	1.5863 (17)	C26—S2A	1.6389 (19)
C11—C12	1.5863 (17)	C26—C27	1.6389 (19)
C11—C12A	1.6815 (15)	C26—C27A	1.6695 (17)
C11—S1	1.6815 (15)	C26—S2	1.6695 (17)
C12—C13	1.540 (2)	C27—C28	1.571 (2)
C12—H12	0.9500	C27—H27	0.9500
S1A—C13	1.540 (2)	S2A—C28	1.571 (2)
C13—C14	1.354 (3)	C28—C29	1.347 (3)
C13—H13	0.9500	C28—H28	0.9500
C14—C12A	1.6288 (19)	C29—C27A	1.605 (2)
C14—S1	1.6288 (19)	C29—S2	1.605 (2)
C14—H14	0.9500	C29—H29	0.9500
C15—O2	1.430 (2)	C30—O4	1.429 (2)
C15—H15A	0.9800	C30—H30A	0.9800
C15—H15B	0.9800	C30—H30B	0.9800
C15—H15C	0.9800	C30—H30C	0.9800
N1—N2	1.3780 (16)	N3—N4	1.3768 (17)
C12A—H12A	0.9500	C27A—H27A	0.9500
			
C6—C1—C2	118.52 (13)	C21—C16—C17	118.26 (14)
C6—C1—C7	120.41 (12)	C21—C16—C22	119.78 (14)
C2—C1—C7	121.07 (13)	C17—C16—C22	121.93 (14)
C3—C2—C1	120.60 (13)	C18—C17—C16	120.85 (14)
C3—C2—H2	119.7	C18—C17—H17	119.6
C1—C2—H2	119.7	C16—C17—H17	119.6
C2—C3—C4	120.31 (13)	C17—C18—C19	120.20 (14)
C2—C3—H3	119.8	C17—C18—H18	119.9
C4—C3—H3	119.8	C19—C18—H18	119.9
O2—C4—C5	125.10 (13)	O4—C19—C20	124.70 (14)
O2—C4—C3	114.90 (12)	O4—C19—C18	115.48 (14)
C5—C4—C3	119.99 (13)	C20—C19—C18	119.82 (14)
C4—C5—C6	119.10 (13)	C19—C20—C21	119.32 (14)
C4—C5—H5	120.5	C19—C20—H20	120.3
C6—C5—H5	120.5	C21—C20—H20	120.3
C1—C6—C5	121.40 (12)	C16—C21—C20	121.46 (14)
C1—C6—H6	119.3	C16—C21—H21	119.3
C5—C6—H6	119.3	C20—C21—H21	119.3
N1—C7—C1	118.66 (12)	N3—C22—C16	119.79 (14)
N1—C7—H7	120.7	N3—C22—H22	120.1
C1—C7—H7	120.7	C16—C22—H22	120.1
N2—C8—H8A	109.5	N4—C23—H23A	109.5
N2—C8—H8B	109.5	N4—C23—H23B	109.5
H8A—C8—H8B	109.5	H23A—C23—H23B	109.5
N2—C8—H8C	109.5	N4—C23—H23C	109.5
H8A—C8—H8C	109.5	H23A—C23—H23C	109.5
H8B—C8—H8C	109.5	H23B—C23—H23C	109.5
O1—C9—N2	121.10 (13)	O3—C24—N4	121.06 (15)
O1—C9—C10	121.10 (13)	O3—C24—C25	121.11 (14)
N2—C9—C10	117.78 (12)	N4—C24—C25	117.80 (13)
C11—C10—C9	110.00 (11)	C26—C25—C24	109.96 (13)
C11—C10—H10A	109.7	C26—C25—H25A	109.7
C9—C10—H10A	109.7	C24—C25—H25A	109.7
C11—C10—H10B	109.7	C26—C25—H25B	109.7
C9—C10—H10B	109.7	C24—C25—H25B	109.7
H10A—C10—H10B	108.2	H25A—C25—H25B	108.2
C10—C11—S1A	122.26 (11)	C25—C26—S2A	123.86 (12)
C10—C11—C12	122.26 (11)	C25—C26—C27	123.86 (12)
C10—C11—C12A	123.80 (11)	C25—C26—C27A	121.30 (12)
S1A—C11—C12A	113.85 (10)	S2A—C26—C27A	114.82 (10)
C10—C11—S1	123.80 (11)	C25—C26—S2	121.30 (12)
C12—C11—S1	113.85 (10)	C27—C26—S2	114.82 (10)
C13—C12—C11	99.52 (10)	C28—C27—C26	96.38 (12)
C13—C12—H12	130.2	C28—C27—H27	131.8
C11—C12—H12	130.2	C26—C27—H27	131.8
C13—S1A—C11	99.52 (10)	C28—S2A—C26	96.38 (12)
C14—C13—C12	116.71 (14)	C29—C28—C27	117.29 (17)
C14—C13—S1A	116.71 (14)	C29—C28—S2A	117.29 (17)
C14—C13—H13	121.6	C29—C28—H28	121.4
C12—C13—H13	121.6	C27—C28—H28	121.4
C13—C14—C12A	115.10 (14)	C28—C29—C27A	116.63 (16)
C13—C14—S1	115.10 (14)	C28—C29—S2	116.63 (16)
C13—C14—H14	122.4	C28—C29—H29	121.7
S1—C14—H14	122.4	S2—C29—H29	121.7
O2—C15—H15A	109.5	O4—C30—H30A	109.5
O2—C15—H15B	109.5	O4—C30—H30B	109.5
H15A—C15—H15B	109.5	H30A—C30—H30B	109.5
O2—C15—H15C	109.5	O4—C30—H30C	109.5
H15A—C15—H15C	109.5	H30A—C30—H30C	109.5
H15B—C15—H15C	109.5	H30B—C30—H30C	109.5
C7—N1—N2	119.49 (11)	C22—N3—N4	119.20 (13)
C9—N2—N1	116.55 (11)	C24—N4—N3	116.90 (13)
C9—N2—C8	120.98 (11)	C24—N4—C23	120.61 (13)
N1—N2—C8	122.28 (11)	N3—N4—C23	122.45 (13)
C4—O2—C15	117.14 (12)	C19—O4—C30	117.02 (13)
C14—S1—C11	94.79 (8)	C29—S2—C26	94.82 (11)
C14—C12A—C11	94.79 (8)	C29—C27A—C26	94.82 (11)
C14—C12A—H12A	132.6	C29—C27A—H27A	132.6
C11—C12A—H12A	132.6	C26—C27A—H27A	132.6
			
C6—C1—C2—C3	−2.5 (2)	C21—C16—C17—C18	−1.8 (2)
C7—C1—C2—C3	177.32 (13)	C22—C16—C17—C18	176.36 (15)
C1—C2—C3—C4	0.1 (2)	C16—C17—C18—C19	−0.6 (2)
C2—C3—C4—O2	−178.41 (13)	C17—C18—C19—O4	−177.30 (14)
C2—C3—C4—C5	2.0 (2)	C17—C18—C19—C20	2.7 (2)
O2—C4—C5—C6	178.77 (13)	O4—C19—C20—C21	177.79 (14)
C3—C4—C5—C6	−1.7 (2)	C18—C19—C20—C21	−2.2 (2)
C2—C1—C6—C5	2.8 (2)	C17—C16—C21—C20	2.3 (2)
C7—C1—C6—C5	−177.01 (12)	C22—C16—C21—C20	−175.90 (14)
C4—C5—C6—C1	−0.7 (2)	C19—C20—C21—C16	−0.3 (2)
C6—C1—C7—N1	172.62 (13)	C21—C16—C22—N3	171.72 (14)
C2—C1—C7—N1	−7.2 (2)	C17—C16—C22—N3	−6.4 (2)
O1—C9—C10—C11	92.19 (16)	O3—C24—C25—C26	−93.93 (18)
N2—C9—C10—C11	−86.14 (16)	N4—C24—C25—C26	84.33 (17)
C9—C10—C11—S1A	−82.95 (15)	C24—C25—C26—S2A	−116.66 (14)
C9—C10—C11—C12	−82.95 (15)	C24—C25—C26—C27	−116.66 (14)
C9—C10—C11—C12A	93.33 (14)	C24—C25—C26—C27A	61.63 (16)
C9—C10—C11—S1	93.33 (14)	C24—C25—C26—S2	61.63 (16)
C10—C11—C12—C13	178.21 (12)	C25—C26—C27—C28	−179.91 (14)
S1—C11—C12—C13	1.59 (11)	S2—C26—C27—C28	1.71 (13)
C10—C11—S1A—C13	178.21 (12)	C25—C26—S2A—C28	−179.91 (14)
C12A—C11—S1A—C13	1.59 (11)	C27A—C26—S2A—C28	1.71 (13)
C11—C12—C13—C14	−0.83 (16)	C26—C27—C28—C29	−2.45 (19)
C11—S1A—C13—C14	−0.83 (16)	C26—S2A—C28—C29	−2.45 (19)
S1A—C13—C14—C12A	−0.25 (19)	S2A—C28—C29—C27A	2.5 (2)
C12—C13—C14—S1	−0.25 (19)	C27—C28—C29—S2	2.5 (2)
C1—C7—N1—N2	−179.76 (11)	C16—C22—N3—N4	−177.97 (13)
O1—C9—N2—N1	−177.36 (13)	O3—C24—N4—N3	−176.37 (14)
C10—C9—N2—N1	0.97 (18)	C25—C24—N4—N3	5.4 (2)
O1—C9—N2—C8	−2.3 (2)	O3—C24—N4—C23	1.5 (2)
C10—C9—N2—C8	176.08 (12)	C25—C24—N4—C23	−176.75 (13)
C7—N1—N2—C9	176.67 (12)	C22—N3—N4—C24	178.08 (13)
C7—N1—N2—C8	1.63 (19)	C22—N3—N4—C23	0.2 (2)
C5—C4—O2—C15	−0.3 (2)	C20—C19—O4—C30	−2.7 (2)
C3—C4—O2—C15	−179.90 (13)	C18—C19—O4—C30	177.30 (15)
C13—C14—S1—C11	1.12 (13)	C28—C29—S2—C26	−1.01 (18)
C10—C11—S1—C14	−178.21 (12)	C25—C26—S2—C29	−179.06 (14)
C12—C11—S1—C14	−1.65 (10)	C27—C26—S2—C29	−0.63 (12)
C13—C14—C12A—C11	1.12 (13)	C28—C29—C27A—C26	−1.01 (18)
C10—C11—C12A—C14	−178.21 (12)	C25—C26—C27A—C29	−179.06 (14)
S1A—C11—C12A—C14	−1.65 (10)	S2A—C26—C27A—C29	−0.63 (12)

### (E)-N'-(4-Methoxybenzylidene)-N-methyl-2-(thiophen-2-yl)acetohydrazide (II) Hydrogen-bond geometry (Å, º)

Cg1, Cg2, Cg3 and Cg4 are the centroids of the C11–C14/S1, C1–C6, C26–C29/S2 and C16–C21 rings, respectively.

**Table:**

*D*—H···*A*	*D*—H	H···*A*	*D*···*A*	*D*—H···*A*
C6—H6···S1^i^	0.95	2.87	3.7326 (14)	152
C10—H10*B*···O3	0.99	2.56	3.5366 (19)	169
C13—H13···O2^ii^	0.95	2.58	3.499 (2)	164
C15—H15*A*···O3^iii^	0.98	2.50	3.478 (2)	176
C25—H25*A*···O1	0.99	2.38	3.3206 (19)	157
C28—H28···O4^iv^	0.95	2.42	3.307 (2)	156
C29—H29···O1^v^	0.95	2.50	3.422 (2)	163
C30—H30*A*···O1^vi^	0.98	2.45	3.419 (2)	168
C6—H6···*Cg*1^i^	0.95	2.67	3.6071 (15)	169
C8—H8*C*···*Cg*2^i^	0.98	2.72	3.4831 (16)	135
C21—H21···*Cg*3^vii^	0.95	2.90	3.6721 (18)	140
C23—H23*A*···*Cg*4^vii^	0.98	2.81	3.6560 (16)	145
C23—H23*C*···*Cg*4^viii^	0.98	2.88	3.6067 (16)	131

Symmetry codes: (i) −*x*+2, −*y*+1, −*z*; (ii) −*x*+2, −*y*+2, −*z*; (iii) −*x*+1, −*y*+2, −*z*; (iv) −*x*+1, −*y*, −*z*+1; (v) *x*−1, *y*, *z*; (vi) −*x*+2, −*y*, −*z*+1; (vii) −*x*+1, −*y*+1, −*z*+1; (viii) −*x*+2, −*y*+1, −*z*+1.
